# Fibroblast-like synoviocyte metabolism in the pathogenesis of rheumatoid arthritis

**DOI:** 10.1186/s13075-017-1303-3

**Published:** 2017-05-31

**Authors:** Marta F. Bustamante, Ricard Garcia-Carbonell, Katrijn D. Whisenant, Monica Guma

**Affiliations:** Department of Medicine, School of Medicine, UCSD, 9500 Gilman Drive, La Jolla, CA 92093-0663 USA

**Keywords:** Fibroblast-like synovicyte, FLS, Metabolism, Glycolysis, Bioactive lipids

## Abstract

An increasing number of studies show how changes in intracellular metabolic pathways alter tumor and immune cell function. However, little information about metabolic changes in other cell types, including synovial fibroblasts, is available. In rheumatoid arthritis (RA), fibroblast-like synoviocytes (FLS) are the most common cell type at the pannus–cartilage junction and contribute to joint destruction through their production of cytokines, chemokines, and matrix-degrading molecules and by migrating and invading joint cartilage. In this review, we show that these cells differ from healthy synovial fibroblasts, not only in their marker expression, proto-oncogene expression, or their epigenetic changes, but also in their intracellular metabolism. These metabolic changes must occur due to the stressful microenvironment of inflamed tissues, where concentrations of crucial nutrients such as glucose, glutamine, and oxygen are spatially and temporally heterogeneous. In addition, these metabolic changes will increase metabolite exchange between fibroblast and other synovial cells, which can potentially be activated. Glucose and phospholipid metabolism as well as bioactive lipids, including sphingosine-1-phosphate and lysophosphatidic acid, among others, are involved in FLS activation. These metabolic changes likely contribute to FLS involvement in aspects of immune response initiation or abnormal immune responses and strongly contribute to joint destruction.

## Background

Fibroblast-like synoviocytes (FLS), also known as synovial fibroblasts or type B synoviocytes, are the predominant cell type comprising the structure of the synovial intima. They are organized in two to three layers of cells and constitute 75–80% of all synoviocytes in normal human synovium [1–3], but also in other species like rabbit and mouse [4, 5]. FLS interact with each other and with the extracellular matrix (ECM) via different molecules, including α1β1 integrin, α2β1 integrin [6], and cadherin-11, a calcium-dependent adhesion molecule, which creates a normal appearing lining [7]. In between, synovial macrophages or type A synoviocytes are located in this stromal cell network [8]. FLS were recently proven to be an essential factor in the formation of a normally organized synovial lining [8]. They have an intrinsic capacity to establish a three-dimensional complex synovial lining architecture characterized by the multicellular organization of the compacted synovial lining and the production of synovial fluid (SF) constituents [8].

**Fig. 1:**
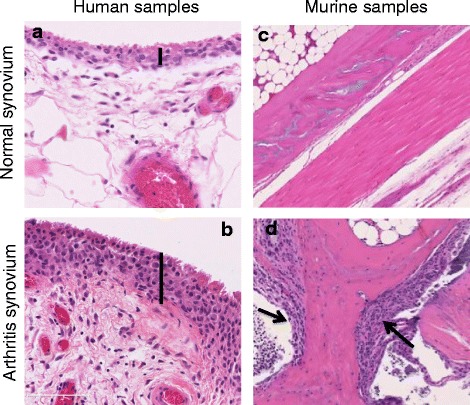
Representative hematoxylin and eosin staining of (**a**) normal synovium lining, (**b**) rheumatoid arthritis synovium showing lining hypertrophy, (**c**) normal bone/synovium interphase in normal murine ankle, (**d**) arthritic bone/synovium interphase in arthritis murine ankle. *Vertical lines* show thickness of synovial lining. *Arrows* indicate pannus that migrated into the bone. Scale bars repressent 100μm

In the inflamed rheumatoid synovium, the healthy two- to three-layer lining structure is converted into a pannus-like structure, a hyperplastic synovial lining containing a higher number of activated FLS and macrophages that extends into the joint space, attaches to the cartilage surface (cartilage–pannus junction), and invades and degrades the cartilage matrix promoting joint destruction (Fig. 1). The sublining layer contains proliferating blood vessels and is invaded by inflammatory cells such as lymphocytes, plasma cells, and macrophages. However, treatments that deplete or reduce these immune populations do not always correlate with better control of the disease. In this regard, targeting chemokines or chemokine receptors to reduce macrophage infiltration in the sublining has failed to show robust clinical improvement [9]. Treatment with the anti-CD20 monoclonal antibody rituximab, which significantly decreases synovial B cells after treatment, did not strongly correlate with clinical response [10]. Yet, cadherin-11-deficient mice have a hypoplastic synovial lining and show a disorganized synovial reaction to inflammation with great resistance to the induction of arthritis and synovium hypertrophy, suggesting a critical role of FLS in synovium architecture in both healthy and arthritic synovial tissue [7].

We would like to further emphasize the unmet need to target FLS in combination with current and future therapies in rheumatoid arthritis (RA). Since Fassbender observed that aggressive resident synovial cells invaded and destroyed cartilage and bone in the joints of patients with RA [11], published data suggest that synoviocytes not only strongly respond to a pro-inflammatory environment, but also actively and autonomously drive joint inflammation and destruction. Among animal models, MRL-lpr/lpr mice that spontaneously develop RA-like symptoms have synovial cells that appear to be activated, attaching to and invading joint structures before inflammatory cells migrate into the synovium [12]. In addition, in a collagen-induced arthritis model, analysis of articular tissues at earlier times showed abnormalities in the synovium, including fibrin deposition in the joints and synovial lining hyperplasia that precedes clinical arthritis [13, 14]. Similarly, examination of the synovial tissues of mice immunized with type II collagen and Complete Freund’s adjuvant (CFA) 10 days before any clinical evidence of joint swelling or tenderness showed hyperplasia of the lining, vasodilation, and mesenchymal-appearing cells, but no infiltration with lymphocytes or leukocytes [15]. Supporting cellular activation in the earliest stage of murine collagen-induced arthritis and rat adjuvant-induced arthritis, the activated form of NF-κB was detected in synovial lining cells 10 days before joint swelling [16]. Although these studies do not prove that FLS drive the disease or are the only activated cell type in the synovium, as crosstalk between synovial fibroblasts and resident macrophages might also play a role in FLS activation, inflammation-independent activation of FLS was confirmed by studies in the severe combined immunodeficiency (SCID) mouse model of cartilage destruction. In these studies, cultured human FLS and also fibroblast-like cells isolated from SF of patients with RA, but not those with osteoarthritis (OA), were co-implanted with human cartilage and found to deeply invade the co-implanted cartilage and the implanted cartilage at a distant site [17, 18].

In RA patients, the number of FLS together with the number of T cells were immunohistological markers in synovial tissue of early RA patients associated with unfavorable disease outcome, suggesting a pathogenic role for these cells in the process of joint damage [19]. The lack of consistent and robust clinical improvement after immune cell targeted therapies also suggests that other synovial cells, and not only immune cells, contribute to chronic inflammation and also joint destruction in RA. This hypothesis is also supported by the recent finding that, in a subgroup of patients considered to have reached clinical remission based on a Disease Activity Score lower than 1.6, joint damage continued to worsen, although low-grade inflammation could not be completely excluded [20]. Together, these findings suggest FLS play a strong role in joint damage and chronic inflammatory-independent FLS activation likely occurs in at least a subset of patients.

## Metabolic changes after FLS activation

**Fig. 2:**
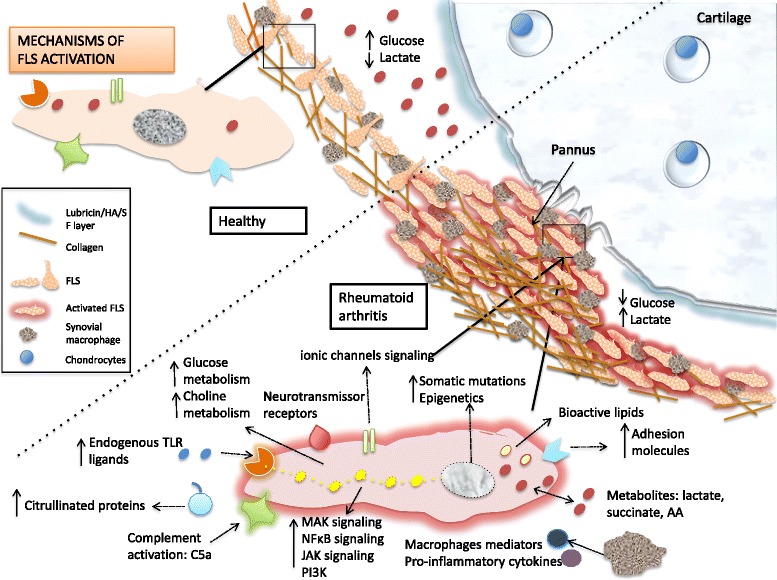
Main mechanisms of FLS activation. The pro-inflammatory environment in RA joints, including high levels of cytokines, growth factors, and infiltrating inflammatory cells, strongly activates FLS. Other stimuli, such as danger-associated molecular patterns (DAMPs), microparticles, activation of calcium channels or stimulation through synovial nerves, complement, and antibodies are also important triggers of inflammatory signaling pathways, metabolic shifts, and epigenetic changes. *AA* amino acids, *PI3K* phosphoinositide 3-kinase, *TLR* Toll-like receptor

Multiple stimuli activate FLS in the disease initiation, perpetuation, and terminal joint destruction phases (Table 1 and Fig. 2). In the initiation phase, initial stimuli such as danger-associated molecular patterns (DAMPs), microparticles, activation of calcium channels or stimulation through synovial nerves, synovial citrullination, complement, and antibodies are also important in the activation of the FLS. Later, the pro-inflammatory environment in RA joints, including high levels of cytokines, growth factors, and infiltrating inflammatory cells, further strongly activates FLS [21]. Furthermore, special environmental conditions in the joints of patients with RA, such as high pressure and hypoxic conditions [22], induce changes that also play a role in FLS activation and aggressive phenotypes.

**Table 1:** FLS in disease initiation, perpetuation, and terminal joint destruction

FLS activated via	FLS response
FLS in arthritis initiation	
TLR endogenous ligands Activated complement Autoantibodies or immune complexes Microparticles Citrullination Synoviocyte-like macrophage mediators Mechanical stimulus	Activation markers Changes in metabolism Signaling pathway activation Synovial hyperplasia Increased expression of adhesion molecules
FLS in arthritis perpetuation	
Hypoxia Cytokine receptors Changes in metabolism Bioactive metabolites Signaling pathway activation Adhesion molecules	Cytokine and chemokine release Recruitment of B and T cells and macrophages to sublining Increase of HLA antigen presentation Proteoglycan damage in cartilage surface Mitochondrial compensatory response
FLS in joint destruction	
Immune cell mediators (cytokines, ligands) Epigenetic changes, hypomethylation, hyperacetylation Somatic and mitochondrial mutation Chronic metabolic changes	Tumor-like transformation Attachment to cartilage Migration and invasion MMP production RANKL expression Resistance to apoptosis

*MMP* metalloprotease, *RANKL* receptor activator of nuclear factor kappa-B ligand, *TLR* Toll-like receptor

Most of the stimuli described above that trigger the FLS response activate a specific receptor or channel located on the cell surface or inside the cell which, in turn, activates signaling pathways in FLS [23–26]. Of note, many signaling pathways activated under these conditions will have profound effects on cellular metabolism to support cell growth, differentiation, and survival. These adaptations must be implemented in the stressful and dynamic microenvironment of inflamed tissues. The MAPK and NF-κB pathways, extensively studied in FLS and critical in FLS activation and the aggressive phenotype [23–26], are also required for an array of metabolic events, though they are more relevant in metabolic tissues such as adipose tissue and liver [27, 28]. Other pathways including key regulators of metabolism in a wide variety of cell types have been reviewed elsewhere [29, 30]. For instance, the PI3K pathway, through AKT1 and mTOR signaling and subsequent downstream hypoxia inducible factor 1 (HIF-1) transcription factor activation, is a major determinant of certain metabolic changes. The AMP activated protein kinase (AMPK) pathway is also of importance, as it is often considered a metabolic checkpoint due to its ability to control cell proliferation when activated under energetic stress [31]. While some of these pathways have been shown to decrease FLS invasive properties [32–35], further studies are needed to link them to altered metabolism in FLS.

**Fig. 3:**
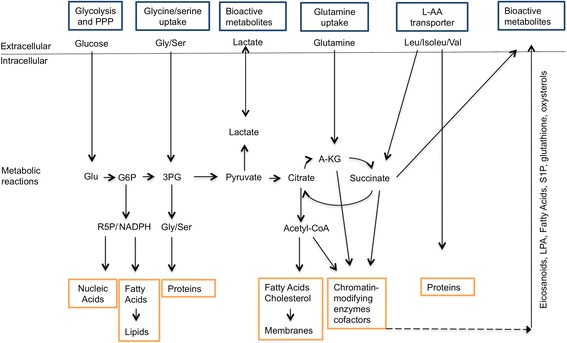
Metabolic alterations involved in activated cells. Activated cells take up large amounts of glucose and glutamine and divert them to the pentose phosphate pathway (*PPP*) and lipid biosynthesis, respectively. Coupled to increased uptake of glycine, serine, and branched chain amino acids (leucine, isoleucine, and valine), which are required for protein synthesis, this generates sufficient building blocks (nucleic acids, proteins, and membranes) for proliferation. The increased generation of reactive oxygen species requires appropriate levels of antioxidants, most of which originate from the PPP. These metabolic changes generate bioactive metabolites that are secreted, and that also contribute to cell activation. *Abbreviations*: *3-PG* 3-phosphoglycerate, *A-KG* α-ketoglutarate, *CoA* coenzyme A, *G6P* glucose-6-phosphate, *LPA* lysophosphatidic acid, *L-AA* L-amino acids, *R5P* ribose-5-phosphate, *S1P* sphingosine-1-phosphate, *PPP* pentose phosphate pathway

After activation, cells change the metabolism of all four major classes of macromolecules (carbohydrates, proteins, lipids, and nucleic acids) and adopt the specific metabolic signatures required for proper effector function. The shuttling of up-regulated metabolites can also serve as a form of intercellular communication [36, 37]. In addition, mitochondria integrate various metabolic pathways and through this process produce intermediates needed for the synthesis of lipids, steroid hormones, and heme, and contribute to thermogenesis [38]. Several reviews have recently described metabolic and mitochondrial changes in immune and tumor cells [39–44]. However, less information is available on stromal cells, including fibroblasts [45], and their contribution to the pathogenesis of autoimmune diseases [46, 47]. Some of the metabolic changes described in other cells and conditions are summarized in Fig. 3.

Recent studies have addressed metabolic changes in the inflamed joints of RA patients and have shown that glucose metabolism is especially increased. Accelerated glucose metabolism is a hallmark of proliferative and activated cells. Elevated glucose metabolism is also required to provide sufficient amounts of metabolic intermediates to support anabolic processes such as nucleic acid, lipid, and protein synthesis. This up-regulation of glycolysis, resulting in increased glucose consumption, can be observed with clinical imaging. Several studies have shown that fluoro-2-deoxyglucose (FDG), which is taken up by glycolytic cells to form FDG-phosphate and can be detected by positron emission tomography (PET), accumulates in swollen joints [48]. Metabolic profiling of synovial tissue showed altered glucose and also choline metabolism in RA samples [49]. Interestingly, one study showed increase uptake of 11C-choline in inflamed joints [50].

We have only recently begun to understand activated metabolic pathways in RA FLS. A recent study evaluated the characteristic metabolic profiling of RA FLS compared to OA FLS using GC/TOF-MS [51]. Sugar metabolism (glycolysis and pentose phosphate pathway) and amino acid metabolism (tyrosine and catecholamine biosynthesis and protein biosynthesis) were severely disturbed in RA FLS compared to those in OA FLS [51]. In vitro studies have also addressed some of these metabolic pathways and metabolite exchange in FLS after activation.

### Glucose and mitochondrial intermediate metabolites

Metabolomics studies in inflammatory arthritis, including RA, suggest an alteration of glycolysis metabolism given the increase of lactate in serum [52]. We evaluated whether FLS glucose metabolism could play a role in inflammation and joint damage [53]. FLS stimulation with tumor necrosis factor (TNF) or platelet derived growth factor (PDGF) increased glucose metabolism and glucose transporter 1 (GLUT1) expression. GLUT1 expression also correlated with FLS baseline functions. Of interest, glucose deprivation or glycolytic inhibitors such as 2-deoxy-D-glucose and bromopyruvate (BrPa) impaired cytokine secretion, proliferation, and migration, and glycolytic inhibition by BrPa administered in vivo in a serum transfer animal model significantly decreased arthritis severity [53].

In the same line, another group showed that hypoxia promoted a switch to glycolysis, supporting abnormal angiogenesis, cellular invasion, and pannus formation [54]. Another glycolytic inhibitor, 3-(3-pyridinyl)-1-(4-pyridinyl)-2-propen-1-one, also significantly inhibited FLS invasion/migration, angiogenic tube formation, secretion of proinflammatory mediators, and activation of HIF1α, pSTAT3, and Notch-1IC under normoxic and hypoxic conditions [54]. Zhao et al. [55] also found an increase of glycolytic genes in collagen-induced arthritis and human synovial and blood samples from RA patients, showing the importance of PGK1 in the pathogenesis of the disease. Thus, the increase of lactate observed in synovial tissue could be secondary to the glycolytic switch in FLS. Of interest, although work on T cells in RA has shown impaired glycolytic flux in these cells due to up-regulation of glucose-6-phosphate dehydrogenase [56], this metabolic reprogramming does help T cells to proliferate and differentiate toward proinflammatory T cells through insufficiently activating the redox-sensitive kinase ataxia telangiectasia mutated (ATM) [57], suggesting that each cell type requires specific metabolic changes to become pathogenic in a particular disease.

Glucose and other metabolites such as acetyl coenzyme A (acetyl-CoA) are then metabolized through the mitochondrial tricarboxylic acid (TCA) cycle in highly metabolic tissues, but a shift in metabolism away from oxidative phosphorylation towards aerobic glycolysis has also been described in inflamed tissues [58]. Interestingly, roles outside metabolism have emerged for intermediates of glucose metabolism and mitochondrial TCA. For instance, lactic acid, the end product of glycolysis, triggered FLS invasiveness [54]. Succinate is abundant in SF from RA patients [59] and critical for angiogenesis [60]. It also stabilizes the transcription factor HIF-1α in activated macrophages and rat synovial fibroblasts [61, 62]. GPR91-deficient mice, which lack a succinate receptor, show reduced macrophage activation and production of IL-1β in an animal model of arthritis [59]. Finally, succinate and other metabolites, including α-ketoglutarate, fumarate, and acetyl-CoA, are involved in changes in the epigenome [61, 63]. These metabolic intermediates might be expected to accumulate in FLS under hypoxic conditions.

### Choline kinase

The choline pathway is highly activated in FLS. An increased level of choline kinase (ChoKα), the enzyme that catalyzes the first step in the CDP-choline pathway and is essential for phosphatidylcholine (PC) expression, has been associated with malignant transformation, invasion, and metastasis in some human cancers [64–67]. ChoKα is also expressed in RA synovial tissue and in cultured FLS [68]. TNF and PDGF stimulation increased ChoKα expression and levels of PC in FLS, suggesting activation of this pathway in the RA synovial environment. A ChoKα inhibitor suppressed the aggressive behavior of cultured RA FLS, including cell migration and resistance to apoptosis. In a K/BxN serum transfer arthritis model, pharmacologic ChoKα inhibition significantly decreased arthritis in pre-treatment protocols as well as in established disease. Interestingly, metabolomics studies correlated choline levels with inflammation in RA [52]. Related to PC, phospholipase D (PLD) enzymes specifically cleave PC, producing phosphatidic acid (PA) and choline. Agonist-induced PLD activation results in PA synthesis, which is thought to be involved in a variety of rapid cellular responses such as cytokine secretion [69]. Several reports suggest a pro-inflammatory role for this enzyme, suggesting that the effects observed after ChoKα inhibition could partially be PA-dependent [70]. The key role of PLD enzymes in basal, IL-17-, and/or TNF-dependent expression of proinflammatory cytokines and chemokines by RA FLS was recently investigated. PLD1-specific siRNAs and small molecule inhibitors specific for PLD1 or PLD2 caused a robust decrease in FLS secretion of IL-6, IL-8, and CCL20, especially when both PLD isoforms were inhibited. Moreover, RA synovial biopsy explants cultured in media containing PLD isoform-specific inhibitors showed significantly reduced constitutive secretion of IL-6 and IL-8 [70].

### Bioactive lipids

Sphingosine kinase (SphK) phosphorylates sphingosine into sphingosine-1-phosphate (S1P). S1P is a well-known bioactive lipid, which has been involved in the pathogenesis of several autoimmune diseases [71]. SphK blockade suppressed cytokines and MMP-9 release in RA peripheral blood mononuclear cells. In addition, downregulation of SphK1, through either a specific siRNA approach or transgenic human TNF SphK1-deficient mice, resulted in significantly less synovial inflammation and joint pathology [72, 73]. Of interest, synovium and SF of RA patients exhibits significantly higher levels of S1P than their non-inflammatory OA counterparts [74].

Another study showed that activated inflammatory arthritis SF from humans and animal models expresses significant quantities of autotaxin (ATX), a lysophospholipase D that catalyzes the conversion of lysophosphatidylcholine (LPC) to lysophosphatidic acid (LPA). ATX expression from SF was induced by TNF, and LPA induced SF activation and effector functions in synergy with TNF [75, 76]. Conditional genetic ablation of ATX in mesenchymal cells, including FLS, resulted in disease attenuation in animal models of arthritis [77]. Notably, high levels of LPC and low PC/LPC ratios in plasma were shown to represent a reliable measure of inflammation [78].

Eicosanoids are other potent bioactive lipids that regulate FLS biology. Leukotriene B(4) (LTB(4)) is a potent proinflammatory lipid mediator that initiates and amplifies synovial inflammation in the K/BxN model of arthritis [79]. FLS generate sufficient levels of LTB(4) after TNF stimulation. Moreover, LTB(4) (acting via LTB(4) receptor 1) was found to modulate the migratory and invasive activity of FLS in vitro and also promote joint erosion by pannus tissue in vivo, placing LTB(4) regulation of FLS biology at the center of a previously unrecognized amplification loop for synovial inflammation and tissue pathology [79]. Of interest, another eicosanoid, the 15-LOX downstream product of 15-(S)-HETE (15-S-hydroxyeicosatetraenoic acid), increased the mRNA and protein levels of MMP-2 in RA FLS. The enhanced effect of 15-(S)-HETE was antagonized by the addition of LY294002 (PI3K inhibitor) and PDTC (NF-κB inhibitor) [80].

Other lipids, such as free fatty acids (FFA) [81], are not only metabolic substrates but may also directly contribute to articular inflammation and degradation in inflammatory joint diseases. Moreover, the data suggest that, in FLS, FFA exert their effects via TLR4 and require extracellular and intracellular access to the TLR4 receptor complex. Bioactive lipids can also have anti-inflammatory effects. For instance, phosphatidylserine inhibits inflammatory responses in IL-1β-stimulated FLS and alleviates carrageenan-induced arthritis in rats [82].

## Metabolic changes deregulate FLS phenotype after activation

The response after FLS activation involves changes in the expression of genes and results in the acquisition of new functions, such as the production of cytokines, chemokines, and tissue remodeling enzymes, and the ability to proliferate, migrate, and invade cartilage. There is a growing appreciation of the fact that reaching these activated states requires the input of new metabolites into specific pathways and therefore there is a strong interest in how metabolic pathways are regulated to support these functional changes. Very limited data are available in FLS, although we hypothesize that, similar to other cell types, especially similar to tumor cells, metabolic changes would support and direct the FLS aggressive phenotype. We expect that inflammation, hypoxia, and the need to support high-energy-demanding processes such as migration and invasion will modify FLS metabolism. In addition, fibroblasts are also critical for SF synthesis, which increases, requiring energy-demanding biosynthesis. Fighting oxidative stress would increase flux through the pentose phosphate pathway to produce cytoplasmic NADPH, requiring high mitochondrial membrane potential in the conversion of matrix NADH to NADPH. We also envision that, given the low apoptosis rate, these cells will have intact compensatory mitochondrial mechanisms to respond to increased ATP demands, increased oxidative stress, and other stress signals for continued support of cellular functions and survival.

### Deregulation of SF

In RA, SF loses its rheological properties. This implies a loss of lubricant properties, which are poor in RA SF, leading to loss of protective properties [83, 84]. This might be due to changes in the RA SF composition. Lipidomics studies found that RA SF has increased levels of sphingolipids and phospholipids compared to normal SF. In fact, primarily lipids that contain choline, such as phosphocholine, phosphatidilethanolamine, and sphingomielins, are increased in RA SF compared with normal SF [85, 86]. These lipid compounds are likely formed in the lamellar bodies of FLS [87]. Although phospholipids are increased in RA, they have shorter chains [88], which are less effective in decreasing friction [89]. Lubricin levels in RA SF are also decreased, and the molecular weight of hyaluronic acid is lower in RA SF [88], which is less effective for SF lubricity, turning it into a pro-inflammatory signal via the TLR4/Myd88 pathway [90, 91]. RA SF is acidic due to high lactate [53, 92, 93] and has a lower glucose content than normal SF [94], likely secondary to an increase of glucose uptake from synovial membrane as suggested by FDG PET images [95]. Given that FLS synthesize numerous cell-surface, ECM, and SF glycoproteins that are required for maintenance of the joint, it would be of interest to know how glucose or other nutrients and metabolic changes affect the biosynthesis of glycoproteins and SF properties [96].

### Deregulation of angiogenesis

Hyperplasia of FLS leads to over-proliferation of synovial tissue, resulting in increased oxygen consumption in synovium, thereby forming a hypoxic environment. The main mechanism that promotes the development of new vessel formation is hypoxia [97, 98]. Hypoxic state leads to the activation of HIF, which activates the expression of HIF-responsive genes, including vascular endothelial growth factor (VEGF), which are important in synovium angiogenic processes and RA perpetuation [99]. VEGF and other growth factors critical for angiogenesis, such as angiopoetin-2, placental growth factors, or fibroblast growth factors, are secreted by FLS and activated synovial macrophages. Importantly, hypoxia stimulates glycolysis and certain glycolytic enzymes, including glucose-6-phosphate isomerase (G6PI) [100], and glycolytic intermediates such as lactate [54] and succinate [60] can be secreted outside the cells and are potent angiogenic stimuli, thus perpetuating angiogenesis.

### Attachment, migration, and invasion of cartilage

RA FLS not only become hyperplastic but also increase their migration and mobility, invading the cartilage and destroying it. Despite a growing understanding of the clinical course of RA, early phases of the disease and the specific sequences of cellular events and interactions leading to progressive destruction of articular structures have not been clarified. In vitro studies suggest that damaged cartilage facilitates the attachment of cells, but there is also evidence that the primary attachment of FLS to matrix components such as collagens and proteoglycans contributes to activation of signaling pathways, triggering their invasive behavior. Thus, a variety of integrins, especially those of the β1 family, have been found to be overexpressed in RA FLS, and blocking these integrins on the surface of RA FLS reduces their attachment and invasive capacity [101–103].

The reciprocal regulation of integrin-dependent functions and cell metabolism is an emerging paradigm [104]. Metabolic pathways such as AMPK, mTORC1, and HIF regulate integrin function on many levels, including regulation of transcription, membrane traffic, and degradation. Moreover, metabolic flux through specific pathways directly remodels integrin function by controlling the integrin glycan profile or integrin structure [104]. In turn, integrins and integrin-derived signals control metabolic pathways, either through engagement of specific signaling pathways or by direct association with metabolic enzymes such as membrane transporters. For instance, β1 integrin interacts with CD98, a protein involved in amino acid transport; increased amino acids levels activate mTORC1 [105]. For instance, increased glucose metabolism in tumor cells is promoted by Twist through a β1-integrin/FAK/PI3K/AKT/mTOR pathway [106].

Several molecules and growth factors, such as PDGF, are essential for the formation of ECM-degrading invadosomal structures and cartilage invasion and activate several signaling pathways, including the PI3K/AKT pathway, known to be upstream of glucose metabolism [107]. Also, FLS secrete collagenases and metalloproteases (MMP), such as MMP-9, that start degrading cartilage ECM after activation [108]. Other MMP involved are MMP-13 [109], MMP-1 [110], MMP-2 [111], and MMP-3. The intimal lining is the major source of MMPs in RA, and in situ hybridization studies localize collagenase messenger RNA almost exclusively to FLS. As described above, the expression of some of these MMP (MMP-1 and MMP-3) in FLS in vitro is modified by glucose or choline metabolism inhibition [53, 68]. Finally, as in angiogenesis, some of the glycolytic enzymes, including glucose-6-phosphate isomerase (G6PI) [100] and glycolytic intermediates such as lactate and succinate [54], induce FLS invasion.

### Apoptosis versus proliferation

The mechanisms of intimal lining hyperplasia remain controversial. While active proliferation is one explanation, and some proto-oncogenes, such as c-myc, are expressed in FLS, the lack of mitoses and absence of immunohistochemical staining for some nuclear proliferation markers suggest that local cell division might not significantly contribute to the increased number of cells.

#### Resistance to apoptosis

Resistance to programmed cell death (apoptosis) induced by the apoptotic stimulus signals, which are abundant in the inflamed joint, is a prominent characteristic of RA FLS. Apoptosis is a mechanism by which cells undergo death to control cell proliferation or in response to DNA damage. RA FLS exhibit changes in mitochondrial pathways of apoptosis and are resistant to receptor-mediated apoptosis at multiple levels. Several explanations have been proposed, including deregulation of the Bcl-2 family of proteins critical to intrinsic pathway regulation [112, 113], deregulation of the NF-κB signaling pathway [114], p53 mutations, and low expression of PUMA, were found in RA synovium and FLS, which provides an explanation for the lack of p53-induced FLS apoptosis [115, 116]. The role of metabolism on apoptosis and mitochondrial response in other cell types, especially tumor cells, has been extensively studied [117, 118]. Among other mechanisms, essential glycolytic enzymes can be translocated into the nucleus, where they play roles independent of their canonical metabolic roles, including anti-apoptosis, providing a possible link between metabolism and apoptosis. For instance, hexokinase 2 (HK2), which phosphorylates glucose to produce glucose-6-phosphate, binds to the mitochondrial membrane via its interaction with the outer membrane porin protein voltage-dependent anion channel (VDAC) [118]. The interaction between HK2 and VDAC inhibits the release of intermembrane pro-apoptotic proteins, thereby protecting cells from apoptosis. Of interest, the expression of HK2 is increased in RA FLS compared to OA FLS [53], and might play a role in FLS apoptosis resistance. Also, RA FLS have a higher glycolytic rate with increased anaerobic respiration and lactate production. This lactate acidifies the ECM, an event that protects it from apoptosis through the regulation of calcium mobilization in capsaicin-induced apoptosis in synoviocytes [119].

#### Altered autophagy

Autophagy is an endogenous process necessary for the turnover of organelles, maintaining cellular homeostasis and directing cell fate. Mitophagy or autophagy of mitochondria is required to eliminate dysfunctional mitochondria and is critical to maintain appropriate metabolic and cell survival signals. Increased mitophagy or autophagy provides survival advantage to the cells in nutrient-deprived or hypoxic conditions. In fact RA FLS showed an increase of genes involved in autophagy, such as beclin-1 and LC3, which inversely correlates with their apoptosis rate [120]. In addition, RA FLS under endoplasmic reticulum stress might resist against apoptosis increasing autophagy [121]. However, we lack information regarding whether or not RA FLS mitochondria have up-regulated some compensatory mitochondrial mechanisms, including mitophagy, to further resist against apoptosis. Further studies are needed to understand the tangled relationship between mitochondria, apoptosis, and autophagy in FLS.

### Crosstalk with synovial cells

No cell type explains the pathologic behavior of RA synovial tissue. Rather, it is the interactions between these cells that define the disease. These various cell types can interact in two general ways: first, through secreted mediators and through direct cell–cell contact that is mediated by cell surface receptors and ligands. Metabolic changes will modify metabolite exchange between cells and can potentially help in RA chronic inflammation. As mentioned above, intermediates of glucose metabolism and mitochondrial TCA have extracellular functions, including angiogenesis, invasiveness, and antiapoptosis [122]. Amino acids, including branched amino acids, have been shown to affect cell signaling (e.g., mediating the mTOR pathway) [123]. Yet another example is kynurenine, a tryptophan metabolite generated by indoleamine 2,3-dioxygenase (IDO) which stimulates TH2 polarization and Treg cell recruitment [124]. Finally, glutathione released by dendritic cells is cleaved to cysteine, which promotes effector T cell proliferation [125]. Lipid signaling can occur via activation of a variety of receptors, including G protein-coupled and nuclear receptors, and members of several different lipid categories have been identified as signaling molecules and cellular messengers [126]; examples include sphingosine-1-phosphate, the inositol phosphates derived from the phosphatidylinositolphosphates (PIPs), diacylglycerol, short fatty acids, eicosanoids, and the oxysterols, such as 25-hydroxy-cholesterol, which are liver X receptor agonists.

In arthritic synovium, although no specific co-culture studies have been described, some of the above-mentioned intermediate metabolites that stimulate FLS, including succinate or lactate, are known to be secreted by cells present in the synovium like macrophages and might work through both autocrine and paracrine mechanisms. Further studies are needed to know whether the above or other mechanisms play a role in the crosstalk between FLS and other synovial cell types.

### Epigenetics

Epigenetic changes are changes in gene function that are heritable over generations without altering the DNA sequence. Short-lived chromatin modifications can also substantially alter gene function and indirectly initiate heritable epigenetic changes of the genome. There are three main mechanisms—DNA methylation, histone modification, and expression of microRNAs—with significant cross-regulation [127]. Epigenetic mechanisms recently raised great interest in the study of the pathogenesis of chronic diseases [128–131]. Accumulating data suggest that epigenetic changes in stromal cell populations might be crucially involved in RA pathology, as epigenetic mechanisms are involved in FLS aggressive phenotype [127, 132]. The reciprocal interaction between metabolism and epigenetics is an emerging field of study; there is a dynamic relationship between metabolic processes and gene regulation via the remodeling of chromatin. Most chromatin-modifying enzymes use cofactors, which are products of metabolic processes. For instance, cofactors such as succinate, nicotinamide adenine dinucleotide (NAD), acetyl-CoA, S-adenosyl methionine, α-ketoglutarate, or flavin adenine dinucleotide (FAD), have critical roles in regulating chromatin processes [133]. Emerging evidence suggests that environmental factors, including lifestyle and diet, might cause epigenetic changes resulting in long-term changes in organ function. For instance, choline, the plasma levels of which correlate with dietary amount, has been implicated as an epigenetic modifier of the genome that may alter gene methylation, expression, and cellular function [134]. Over the past decade, multiple studies have also shown that nutrition can alter the metabolic phenotype of offspring, raising the question of how metabolism regulates the epigenome of germ cells [135].

Conversely, some epigenetic factors have been shown to regulate metabolic genes leading to a shift in energy flow. For instance, emerging evidence now indicates that microRNAs play a significant role in regulating the expression and activity of peroxisome proliferator-activated receptors (PPARs), which control the transcription of genes involved in energy homeostasis [136]. MicroRNAs also regulate expression of genes involved in several aspects of cell metabolism, including glycolysis and the mitochondrial TCA cycle, and were shown to have a major impact on signal transduction via PI3K/AKT and HIF1 [137, 138]. DNA methylation and histone modifications were also shown to modulate the expression of metabolic genes, including gluconeogenesis enzymes and sirtuins, respectively [139]. A detailed study of the epigenetic–metabolism crosstalk in FLS could explain why RA FLS early in the initiation or later in the chronic phase of the disease might produce high levels of disease-promoting molecules without further stimulation by immune cells.

## Conclusions

Most of the therapies approved for RA do not directly target FLS. Biological therapies that focus on inhibiting immune cell activation or inflammatory cytokines likely improve the inflammation-dependent FLS activation but do not address the chronic inflammatory-independent FLS activation observed in a subset of patients. Reprogramming metabolic changes might be a new modality key to developing joint-protective strategies in RA FLS. In fact, the metabolic rewiring of immune cells has been viewed as a promising source of novel drug targets [37, 140]. Resetting metabolism in these cells as well and in stromal cells contributing to pathogenesis offers novel opportunities for disease modulation in RA. In fact, rheumatologists already use the antimetabolites methotrexate and leflunomide for the treatment of patients with inflammatory arthritis. And both were shown to inhibit FLS functions [141, 142]. Biological therapies including anti-TNF therapies could also address metabolic changes induced by inflammation, although further studies are needed to link their efficacy to altered metabolism in FLS.

Inhibition of key enzymes that regulate activated pathways, including glycolysis (BrPa or PFKFB3 inhibitor) or phospholipid (ChoK inhibitor) metabolism, would be one way to interfere with these metabolic changes and end products such as lactate, succinate, or bioactive lipids. Other ways to modulate cell and specifically mitochondrial metabolism would be by targeting mitochondria via enhancing mitochondrial biogenesis or by encouraging reliance on oxidative phosphorylation instead of glycolysis. AMPK agonists could improve mitochondria biogenesis if needed. Furthermore, understanding whether there are disease-specific changes in metabolism, or activation of specific changes in metabolic pathways such as mTOR or HIF1a, could lead to identification of key regulatory enzymes or factors as new therapeutic targets. Thus, further characterization of the mechanisms linking cellular metabolism and mitochondrial function is needed to obtain clearer insight into the relationship between mitochondrial homeostasis, metabolic changes, and inflammation in FLS. Whether this truly constitutes an option to increase the drug armamentarium in rheumatic diseases beyond biological and kinase inhibitor therapies remains to be determined.

## Abbreviations

AMPK: AMP-activated protein kinase
ATX: Autotaxin
BrPa: Bromopyruvate
ChoKα: Choline kinase
CoA: Coenzyme A
DAMP: Danger-associated molecular pattern
ECM: Extracellular matrix
FDG: Fluoro-2-deoxyglucose
FFA: Free fatty acid
FLS: Fibroblast-like synoviocyte
GLUT1: Glucose transporter 1
HIF: Hypoxia inducible factor
HK2: Hexokinase 2
IL: Interleukin
LPA: Lysophosphatidic acid
LPC: Lysophosphatidylcholine
LTB(4): Leukotriene B(4)
MMP: Metalloprotease
OA: Osteoarthritis
PA: Phosphatidic acid
PC: Phosphatidylcholine
PDGF: Platelet-derived growth factor
PET: Positron emission tomography
PLD: Phospholipase D
RA: Rheumatoid arthritis
S1P: Sphingosine-1-phosphate
SF: Synovial fluid
SphK: Sphingosine kinase
TCA: Tricarboxylic acid
TNF: Tumor necrosis factor
VEGF: Vascular endothelial growth factor
VDAC: voltage-dependent anion channel
